# Evolutionary Constraint Helps Unmask a Splicing Regulatory Region in BRCA1 Exon 11

**DOI:** 10.1371/journal.pone.0037255

**Published:** 2012-05-16

**Authors:** Michela Raponi, Andrew G. L. Douglas, Claudia Tammaro, David I. Wilson, Diana Baralle

**Affiliations:** 1 Human Development and Health, Faculty of Medicine, University of Southampton, Southampton, United Kingdom; 2 Wessex Clinical Genetics Service, Southampton University Hospitals NHS Trust, Southampton, United Kingdom; National Cancer Institute, National Institutes of Health, United States of America

## Abstract

**Background:**

Alternative splicing across exon 11 produces several *BRCA1* isoforms. Their proportion varies during the cell cycle, between tissues and in cancer suggesting functional importance of *BRCA1* splicing regulation around this exon. Although the regulatory elements driving exon 11 splicing have never been identified, a selective constraint against synonymous substitutions (silent nucleotide variations that do not alter the amino acid residue sequence) in a critical region of *BRCA1* exon 11 has been reported to be associated with the necessity to maintain regulatory sequences.

**Methodology/Principal Findings:**

Here we have designed a specific minigene to investigate the possibility that this bias in synonymous codon usage reflects the need to preserve the *BRCA1* alternative splicing program. We report that in-frame deletions and translationally silent nucleotide substitutions in the critical region affect splicing regulation of *BRCA1* exon 11.

**Conclusions/Significance:**

Using a hybrid minigene approach, we have experimentally validated the hypothesis that the need to maintain correct alternative splicing is a selective pressure against translationally silent sequence variations in the critical region of *BRCA1* exon 11. Identification of the *trans*-acting factors involved in regulating exon 11 alternative splicing will be important in understanding *BRCA1*-associated tumorigenesis.

## Introduction

Pathogenic mutations in the *BRCA1* gene are associated with a high risk of breast and ovarian cancer. Women heterozygous for such mutations have a lifetime risk of up to around 80% of developing breast cancer and up to 40% of developing ovarian cancer [1]. The effects of common deleterious mutations, such as exonic insertions and deletions, nonsense substitutions and substitutions at invariant consensus splice sites (AG and GT), are relatively easy to predict. However, the effects of synonymous, translationally silent substitutions on splicing are generally much less well understood and require investigation through functional studies. Such substitutions are called ‘silent’ as they do not directly change the amino acid sequence of the protein. However, these synonymous substitutions (as well as non synonymous substitutions) can still have a deleterious effect at the RNA level by creating or disrupting secondary structures or regulatory sequences. This in turn may alter splicing fidelity, with consequent loss of function or production of new antagonistic protein isoforms.

*BRCA1* is known to undergo alternative splicing of a number of its exons, including the large and functionally important exon 11 [2]. Alternative splicing of exon 11 yields a full length isoform (FL) and also shorter isoforms through use of an alternative intra-exonic splice donor site, D(11q), or through complete skipping of exon 11, D(11). Alternative splicing can also exclude exons 9 and 10 from the mature mRNA, D(9,10). These isoforms maintain the original *BRCA1* open reading frame and allow functional protein production.

The control of the ratio of splicing isoforms produced within a cell requires regulation by *trans*-acting splicing factors that recognise and bind specific pre-mRNA sequences. Their expression changes in cancer as well as in different tissues [3], [4]. Since *BRCA1* isoforms vary in quantity during the cell cycle and within different tissues [5]–[8], including tumour tissue, it is expected that important splicing regulatory elements may be found at critical gene regions (e.g. within exon 11) that allow the binding of relevant splicing factors. Mutations in these sequence elements would disrupt normal splicing and potentially lead to disease. Therefore there would be an evolutionary selection pressure not only against codon-altering mutations but also against synonymous mutations at these sites. This process, known as purifying selection, is thought to explain the bias in synonymous codon usage observed at specific genomic sites across evolutionarily divergent species. The observation of purifying selection at translationally silent sites may therefore reflect the presence of splicing regulatory elements or regions of critical RNA secondary structure [9]–[10].

A bias towards the usage of particular codons, rather than their synonymous counterparts, has previously been reported in a critical region of *BRCA1* spanning the 3′ end of exon 10 and the 5′ end of exon 11 [11]. Several ‘hotspots’ (codons 195, 196, 215, 231, 244 and 313) have been identified around this critical region [11]. These are putative sites of purifying selection with a high ratio of nonsynonymous to synonymous substitutions.

In order to investigate *BRCA1* splicing involving exon 11, we developed a minigene incorporating the whole of *BRCA1* exons 8 to 11 and part of exon 12. Using this hybrid minigene approach, we have experimentally validated the hypothesis that maintenance of correct alternative splicing is the cause of selection against silent sequence variations in the *BRCA1* critical region spanning exon 11.

## Results

### Minigene splicing assay for BRCA1 exon 11

**Figure 1:**
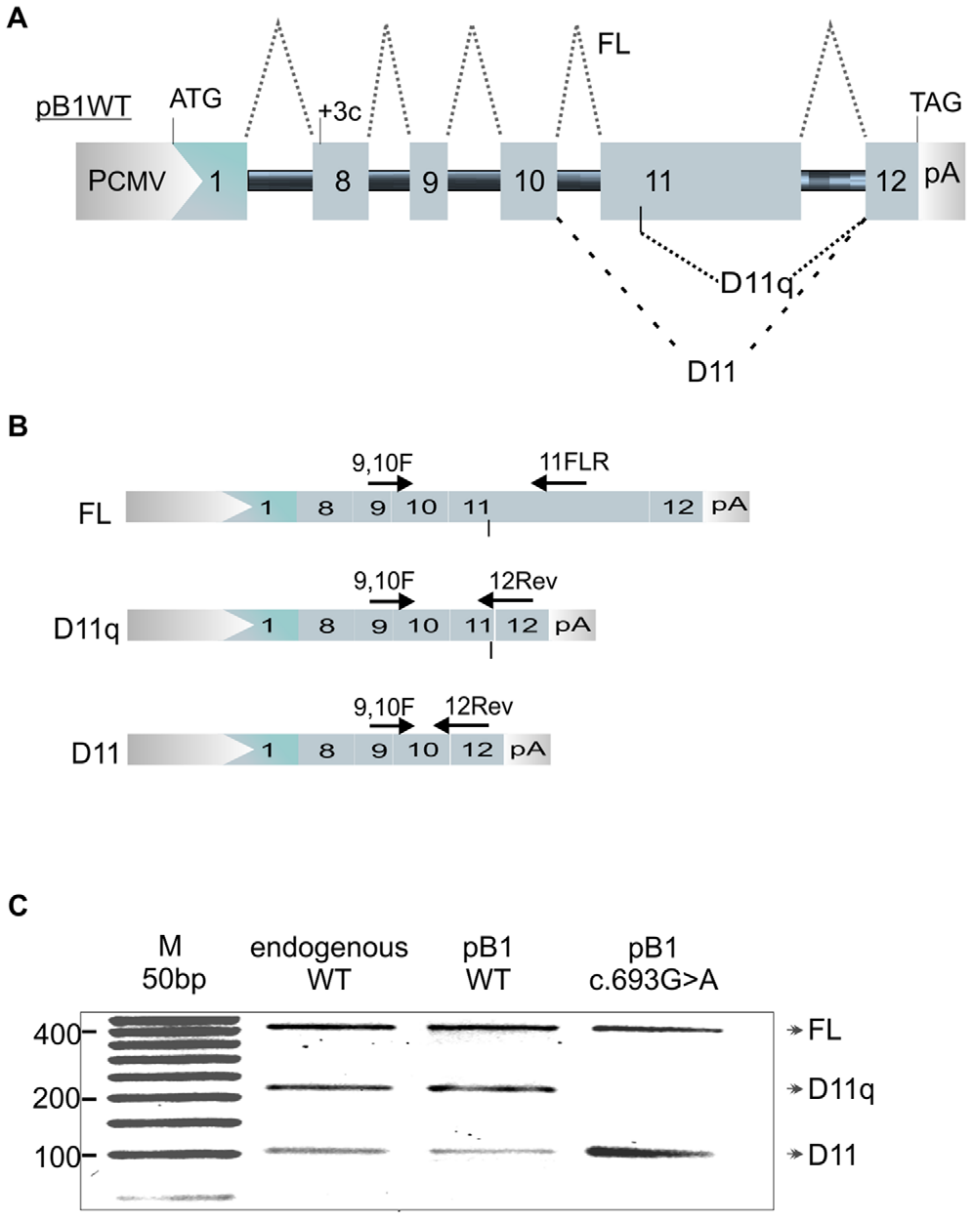
Minigene splicing assay of BRCA1 exon 11. **A**. The pB1 wild type (WT) version of the minigene is shown. PCMV = promoter of the pCDNA3 vector. ATG = start codon. TAG = stop codon. +3C = insertion of cytosine as the third nucleotide in exon 8. pA = poly A signal. 1 = exon 1 of the alfa globin gene. BRCA1 exons from 8 to 12 are numbered. The black solid line represents introns. Dotted lines show alternative splicing of exon 11. **B**. The three splicing isoforms FL, D11q and D11 and the position of specific oligos used for detection, are shown. **C**. Detection of BRCA1 exon 11 splicing isoforms for: _MCF7 endogenous BRCA1 (endogenous WT). _pB1 WT minigene transfected in MCF7 (pB1 WT). _pB1 minigene carrying the c.696G>A nucleotide substitution transfected in MCF7 (pB1 c.696G>A).

In order to assay the effects of synonymous substitutions on *BRCA1* splicing, we constructed a pB1 wild-type minigene that incorporated sequence from *BRCA1* exon 8 up to the first 89 nucleotides of exon 12 (Figure 1a). Intronic sequences were shortened but still contained at least 190 nucleotides of native intronic sequence at either end. Intron 8 is 460 bp (original size 2485 bp); Intron 9 is 379 bp (original size 1321 bp); Intron 10 is 684 bp (original size 985 bp); Intron 11 is 402 bp (original size 402 bp). The pB1 minigene was transiently transfected into MCF7 breast cancer cell lines. RNA was extracted and minigene-specific cDNA synthesised using the specific primer pCSrev. *BRCA1* splicing products were analysed by RT-PCR using primers specific for *BRCA1* FL, D(11) and D(11q) isoforms (Figure 1b). Electrophoresis of RT-PCR *BRCA1* splicing products revealed the presence of the three isoforms FL, D(11) and D(11q); showing comparable outcomes with that of endogenous BRCA1 in non-transfected MCF7 cells (Figure 1c). In order to validate the minigene for splicing assays, we introduced the nucleotide substitution c.696A>G previously reported to affect exon 11 splicing. As shown in Figure 1c, introduction of this change to the minigene caused an increase of the D(11) isoform as described in the literature [12].

The c.696A>G mutated minigene was also tested in normal mammary epithelial cells (HMEpC, invitrogen) and breast cancer cells MDA-MB-231 (ATCC). All cell lines tested resembled the increase in D11 isoform observed in MCF7 cells (data not shown).

Furthermore, a minigene construct containing the genomic sequence from exons 9–12 had the same pattern of splicing Another version of the minigene behaved in exactly the same manner when we inserted the entire genomic sequence from exons 9 to 12 (data not shown).

### Synonymous substitutions of codons thought to undergo purifying selection

**Figure 2:**
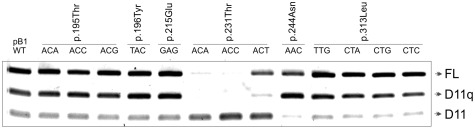
Minigene splicing assay of synonymous substitutions. Effect of synonymous *BRCA1* substitutions on splicing products full-length, D(11q) and D(11). RT-PCR products from transfection experiments using minigenes carrying codon substitution are shown.

Mutations at codons 195, 196, 215, 231, 244 and 313 (which are proposed to exhibit purifying selection) were assayed for their effect on *BRCA1* splicing. 13 synonymous substitutions were introduced by site-directed mutagenesis (Supporting Table S1):

The resulting mutated minigenes were transiently transfected into MCF7 cell lines and splicing analysed as described (Figure 2). Mutations that do not affect splicing would be expected to give a similar isoform band pattern to the wild-type sequence. If splicing of exon 11 is affected, a relative decrease of one isoform should be accompanied by a reciprocal increase in one or more of the other isoforms, or the appearance of a novel isoform. In addition one or more new isoforms could appear.

All three synonymous substitutions at codon 231 appear to alter splicing. These changes lie 23 nucleotides distal to the start of exon 11 and significantly reduce the levels of FL and D(11q) in favour of increased levels of D(11) isoform. This suggests that sequence changes at this site alter the splicing of exon 11, through reduced recognition of the intron 10 acceptor site, resulting in its exclusion from the final mRNA. This finding points towards an important sequence element, such as an exonic splicing enhancer, located around codon 231.

Codon 244 lies 60 nucleotides downstream from the start of exon 11 and is 55 nucleotides proximal to the 11q splice donor site. The T>C synonymous substitution at the third position of this codon seems to enhance levels of the D(11q) isoform. A similar effect was also observed in normal mammary epithelial cells (HMEpC supplied by ECACC) (data not shown).

Codon 313 is situated 150 nucleotides 3′ (downstream) of the 11q splice donor site. Synonymous mutations in this codon do not appear to alter exon 11 splicing. Codons 195 and 196 are 9 and 6 nucleotides away from the 3′ end of exon 9 respectively and synonymous mutations at these sites would therefore most likely affect splicing of exon 9. Codon 215 lies 23 nucleotides from the 3′ end of exon 10 and so any splicing effects may be expected to affect this exon. Our assay does not show any significant alteration in relative exon 11 splice isoform abundance with synonymous mutations at these sites. We therefore evaluated their effect on skipping of exon 9 and 10. Splicing products revealed presence of four isoforms {FL, D(9), D(10) and D(9&10)} showing comparable outcomes with that of the pB1WT minigene (supporting figure S1).

### Other synonymous changes reported in breast cancer patients

Since the c.693G>A substitution at codon 231 affects splicing of *BRCA1* exon 11 and has previously been reported in a patient with breast cancer [12], we decided to evaluate the effects of additional synonymous substitutions in *BRCA1* exon 11 (c.825C>T codon 275, c.828A>G codon 276 and c.795T>C codon 265; supporting table S1) that had been identified in patients with breast cancer ascertained by the local regional genetics service in whom no other pathogenic mutation had been found. After introducing the substitutions into the minigene, MCF7 cell lines were transfected and RNA analysed for FL, D(11q) and D(11) isoforms.

**Figure 3:**
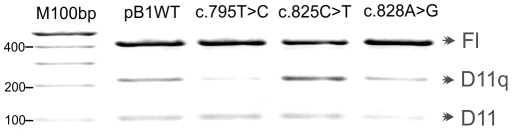
Minigene splicing assay of patients synonymous substitutions. Effect of synonymous *BRCA1* substitutions on splicing products full-length, D(11q) and D(11). RT-PCR products from transfection experiments using mutated minigenes are shown.

The T>C substitution within codon 265 lies just 8 nucleotides 3′ of the 11q splice donor site. Alteration of the native sequence GTAGTTCT to GTAGTTCC results in reduction of the D(11q) isoform (Figure 3 and supporting table S1). This suggests that the sequence at codon 265 influences the use of this alternative splice site. The proximity to the splice donor site makes it likely that the sequence forms part of the splice site itself. The mutation appears to weaken the splice site, decreasing its usage. The synonymous substitution in codon 275 lies 38 nucleotides 3′ of the 11q splice donor site. Its presence appears to increase the relative amount of D(11q) while decreasing the FL isoform but not D(11)(Figure 3 and supporting table S1). This suggests that the substitution favours the usage of D11(q) donor site competing with the FL isoform donor site. The substitution in codon 276 just 3 nucleotides further downstream does not seem to have this same effect. This suggests that codon 276 contains an important sequence element such as an ESE that favours use of the D(11q) splice site. A similar effect on splicing for these three synonymous changes was also observed in HEK 293 cells (data not shown).

D11q intensity seems to be higher in figure 3 than 1 and 2. Although we observed a certain degree of variability between biological replicates (at different transfection dates), changes in isoform proportion (comparing different variants) were consistent. In addition, we did not observe variability between technical replicates (minigene transfected on the same date). This could well be explained by the fact that the BRCA1 isoform proportions vary during the cell cycle [5] so that minimal changes in cell status (e.g. the number of passages) may account for the change in intensity of D11q isoforms between biological replicates.

In addition, these types of effects should be routinely tested with quantitative qRT-PCR methods.

### Targeted deletions of the critical region

In order to identify putative splicing regulatory elements in the ‘critical region’ of *BRCA1* exon 11, we undertook a comparative genomic analysis of the *BRCA1* sequence of 9 eutherian mammals using the Ensembl genome browser (http://www.ensembl.org/index.html). The data show several conserved sequences along the ‘critical region’ (supporting Figure S2). In order to evaluate whether these conserved regions include splicing regulatory sequences we used SFmap (http://sfmap.technion.ac.il/) [13] for the prediction of splicing factor binding sites (supporting Figure S2).

**Figure 4:**
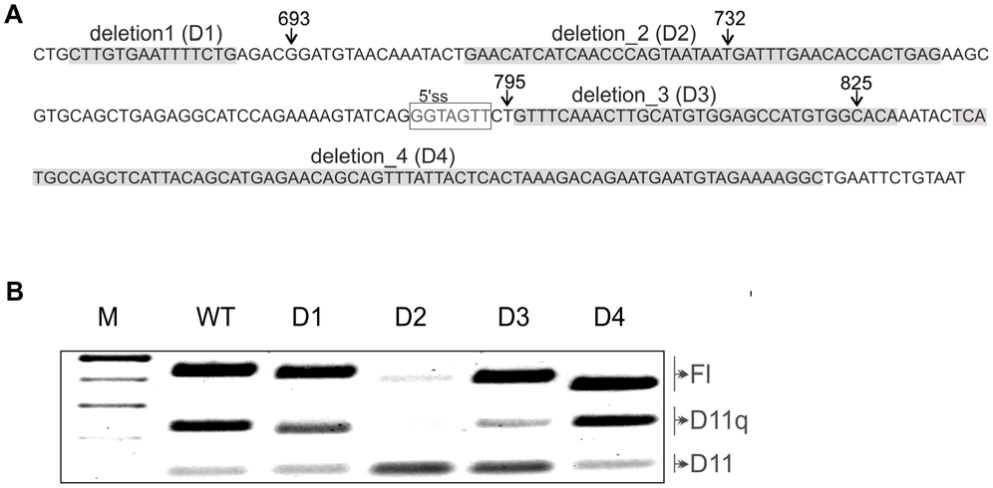
Minigene splicing assay of BRCA1 exon 11. **A**. Sequence of the critical region in exon 11 showing the deletion 1,2,3,4 (highlighted). The donor site (5′ss) generating D11q isoform is boxed. Arrows indicate nucleotide positions of the variations reported in Figure 2 and 3 to affect splicing. **B**. Transient transfection results for the hybrid minigenes carrying deletions.

We then performed a pB1 minigene deletion analysis of the most conserved regions containing putative binding sites for splicing regulatory proteins. The D1, D2, D3 and D4 deletions are highlighted in Figure 4a. Hybrid minigenes carrying each deletion were transiently transfected into MCF7 cells and splicing was analysed (Figure 4b). The results show that *BRCA1* exon 11 splicing is unchanged with deletion D1 and D4. Deletion D3 has a weak effect in reducing levels of the D(11q) isoform in favour of D(11). Transfection with the hybrid minigene carrying deletion D2 has the strongest effect, inducing almost complete skipping of exon 11.

The putative splicing factors predicted to bind in D2 and D3 by SFmap (http://sfmap.technion.ac.il/) and SpliceAid2 (www.introni.it/spliceaid.html) are shown in supporting Figure S3.

## Discussion

That synonymous mutations are under evolutionary constraint due to splicing requirements has been demonstrated experimentally [14]. Hurst and Pal found a pronounced peak in the ratio of non-synonymous to synonymous substitutions between codons 200 and 300 of *BRCA1* exons 10 and 11 when comparing human/dog and mouse/rat gene alignments [11]. The presence of this so-called ‘critical region’ reflects an unusually low rate of translationally silent synonymous sequence changes, suggesting that purifying selection may be acting on this region to select against synonymous changes, possibly in order to preserve splicing regulatory elements. In this study, we have experimentally verified this hypothesis by testing synonymous substitutions at codon sites proposed by Hurst and Pal to have the highest non-synonymous to synonymous substitution ratios.

Of the 6 codons analysed, only synonymous substitutions at codon 231 and codon 244 affected splicing of *BRCA1* exon 11. The substitution at codon 244 increased levels of the D(11q) isoform to the detriment of the full and D(11)isoforms. However, it is difficult to speculate upon the relevance of this aberrant splicing effect. This is for two main reasons. Firstly, the effect on the splicing ratio is only relatively weak. Secondly, the D(11q) isoform has been shown to play a role in apoptosis [15]. However, a recent article has shown that, following knockdown of the nuclear chaperon Ubc9 (ubiquitin conjugating enzyme 9), D(11q) accumulation in the cytoplasm promotes growth and survival of breast cancer cells [16]. This may signify that an increase of D(11q) isoform may have deleterious effects in circumstances where Ubc9 is compromised.

Substitutions at codon 231 caused skipping of exon 11 with a marked increase in amounts of the D(11) isoform. Overexpression of this isoform in mouse epithelial mammary cells has been shown to cause atypical duct hyperplasia [17]. This could explain the biased synonymous codon usage observed at codon 231 and may reflect the necessity to preserve a regulatory sequence that protects against aberrant splicing, which would otherwise predispose to breast cancer.

Substitutions at codons 195, 196, 215 and 313 did not have any discernible effect on isoform levels. However, there may be other ways by which synonymous changes can exert an effect [18]. For instance tRNAs complimentary to different codons vary in their relative concentrations within a cell, meaning that synonymous codons may not be equally represented in terms of the relative abundance of their complimentary tRNAs [19]. This variable cellular availability of specific tRNAs can affect translational efficiency of protein product.

Nevertheless, the possibility that substitutions at these codon positions affect regulatory elements of splicing should not be excluded. In fact protective variants might have occurred during evolution that compensate for aberrant splicing [14], [20].

The c.693G>A substitution at codon 231 has previously been reported to cause exon 11 skipping in a patient with breast cancer [12]. This synonymous change in our minigene gave the same splicing outcome, proving the validity of the pB1 minigene as a splicing assay for *BRCA1* exon 11 variants. We therefore tested three further synonymous variants found in breast cancer patients to investigate their effect on *BRCA1* splicing. Our findings show that the mutations c.795T>C and c.825C>T decrease and increase levels of the D(11q) isoform respectively. However, for the same reasons mentioned above it is not possible to classify these variants as pathogenic mutations causing aberrant splicing since the role of D(11q) is not clear. In fact, the D(11q) isoform has been described as both causing apoptosis and causing cancer [15], [16]. With these opposing roles in mind, we can hypothesise that an increased abundance of this isoform may have competing and contrasting effects in controlling cell proliferation or cell death depending on a patient's personal sequence context.

Both artificial and natural variants analysed in this study cause multiple splicing effects (Supporting Table S1). For instance down-regulation of the D(11q) isoform is not always accompanied by up-regulation of other isoforms. This suggests that different regulatory elements are affected that control one or all of the following events: usage of the exon 11 acceptor site, usage of the exon 11 donor site, usage of the D(11q) isoform donor site, and competition between the two donor sites.

Despite the fact that only synonymous substitutions in 2 out of 6 codons affected exon 11 splicing, the deletion analysis of *BRCA1* exon 11 appears to experimentally validate the hypothesis that maintenance of correct alternative splicing is the cause of selection against silent sequence variations in the *BRCA1* critical region. In fact, 2 out of 4 of these deletions affected the proportion of *BRCA1* splicing isoforms. In summary, deletions 2, and 3 changed *BRCA1* splicing ratios in different and opposing directions.

Deletion 2, in particular, caused almost complete skipping of exon 11 and was predicted to lose splicing regulatory sequences for binding of several splicing enhancer proteins (SC35, SRp20/30/40, NOVA 1 and YB1). Deletion 3, just next to the D(11q) donor site, decreased D(11q) isoform levels, probably due to the loss of a putative binding site for TIA1 (a donor site modulator) predicted by the SpliceAid analysis. NOVA 1 binding was predicted to bind both regions corresponding to deletion 2 and 3. Binding of NOVA1 at the end of an exon and beginning of an intron was proposed to induce exon inclusion [21]. In this case binding of NOVA1 upstream and downstream D11q isoform donor site might regulate production of this isoform.

We did not observe strong correlation on the splicing effect of nucleotide changes occurring inside the region of deletion 2 (c.732T>C) and deletion 3 (c.825C>T). This suggests that composite regulatory elements of splicing might be present in this region of BRCA1 exon 11 as has been previously suggested for CFTR exon 12 [22].

In order to fully understand the effects of altered splice isoform ratios in *BRCA1*-related cancer, it will be necessary to know the roles of each isoform individually and to understand the combined roles of different isoforms both in health and in tumorigenesis. Part of this understanding will require confirmation of which splicing factors are involved in regulating alternative splicing of *BRCA1*, including those suggested by this study that may be involved in the splicing of exon 11. Knowledge of these mechanisms would provide a framework for the development of new therapeutic agents capable of manipulating *BRCA1* splicing and treating *BRCA1*-related cancers (eg. breast ovarian and prostate cancer). At some level it may be that cancer predisposition is not so much down to whether individual isoforms are simply present or absent but rather that subtle alterations to a complex and nuanced isoform profile are particularly relevant. Such an isoform environment may interact with other cellular pathways to make conditions favourable or otherwise towards tumour development. If this is the case, it will provide an added challenge of complexity to our understanding of tumorigenesis. However, it may also allow novel and innovative approaches to manipulating the cellular environment in order to prevent and treat cancer.

## Materials and Methods

### Construction of the minigene

The pB1 WT minigene is shown in Figure 4. It consists of 6 exons (including part of their flanking introns) cloned in a modified version of the pCDNA3(+) vector (Invitrogen) under the control of the CMV promoter. The polylinker of the pCDNA3+ vector has been replaced with an adaptor containing the appropriate restriction sites for cloning of exon 1 of the α-globin gene with its 3′ flanking intronic region together with the relevant *BRCA1* genomic region from exon 8 to exon 12. Introns have been shortened by PCR amplification with oligonucleotides carrying a non complementary tail for specific restriction digestion and subsequent cloning in pCDNA3+.

Exon 1 of the α-globin gene is used as the first exon of the minigene as it provides a strong splice donor site at its 3′ end as well as an ATG start codon at its 5′ end.

Using specific oligonucleotides and a two-step PCR mutagenesis method [23], a stop codon was created in exon 12 and a single nucleotide insertion was created in exon 8 in order to maintain the correct reading frame. Several unique restriction sites are maintained in the sequence in order to facilitate subsequent mutagenesis and deletion analysis. Mutated minigenes and minigenes carrying deletions were generated through a two-step PCR overlap extension [24] using the pB1 WT construct as a template. The identity of all minigenes was checked by sequencing. The minigene complete sequence and oligonucleotidess used for cloning and mutagenesis are available upon request.

### Cell Culture

Human breast cancer cell lines, MCF7 (ATCC number: HTB-22™), were grown in DMEM medium with 4500 mg/L glucose, pyruvate and L-glutamine supplemented with 1% penicillin/streptomycin and 10% fetal bovine serum. Cells were incubated at 37°C in a 5% CO2 atmosphere.

### Transfection

Minigene plasmid vector was transfected into MCF7 cell lines using the FuGENE 6 transfection reagent from Roche. 100 µl of DMEM serum-free medium containing 2 µg of vector DNA and 3 µl of FuGENE reagent was incubated for 15 minutes at room temperature before the mixture was added in 6 cm-well cell cultures (50% confluent) in the presence of 10% fetal bovine serum.

### RNA Extraction and RT-PCR

40 hours after transfection, RNA was extracted from cells using the RNeasy-plus kit from QIAGEN following the manufacturer's instructions. To analyse alternative splicing of *BRCA1* exon 11 in the pB1 minigene, RT-PCR was performed with 1.5 µg of total RNA using the pCSrev primer [5′ GCAACTAGAAGGCACAGTCGAGG 3′] to exclusively target only RNA products from the pB1 minigene. Given the large size of exon 11 (3426 nucleotides), the QIAGEN LongRange RT PCR kit was used following the manufacturer's instructions. 1/4 of the resulting cDNA was amplified in a PCR reaction using primers specific for the desired splice isoforms and PCR products were analysed by gel electrophoresis in 1.5% agarose. The forward primer [9–10F: 5′ ACTTATTGCAGTGTGGGAGA 3′] hybridises to the junction between exons 9 and 10. Reverse primers are a mixture of one specific for FL [11FLR: 5′ GGAGTCCGCCTATCATTACATG 3′] and one specific for D(11q) and D(11) [12R: 5′ CCAGATGCTGCTTCACCCT 3′]. 11FLR hybridises within exon 11 distal to the 11q splice site. 12R hybridises to proximal exon 12 and overlaps the exon 10/12 junction and also the exon 11q/12 junction.

For the analysis of *BRCA1* alternative splicing of exons 9 and 10, RT-PCR was performed on 1 µg total RNA using random primers with the Promega kit. *BRCA1*-minigene specific PCR was performed on resulting cDNA using the forward specific primer alpha-8F [5′ GAGGCCCTGGAGAGGAcAA 3′] and the reverse primer 11+81Rev [5′ TCTCAGTGGTGTTCAAATCA 3′]. Alpha-8F hybridises to the junction between exon 1 of the α-globin gene and exon 8 of *BRCA1*. The c nucleotide in lowercase represents the insertion made in exon 8 of the pB1 minigene. 11+81Rev hybridises to exon 11 upstream of the donor site producing D(11q).

In order to eliminate heteroduplexes from mixed-template 1/10^th^ of PCR products were subjected to ‘reconditioning PCR’ for 6 cycles [25].

### In silico analysis

Putative splicing regulatory sequences in *BRCA1* exon 11 were predicted using the computational tools SFmap [13] and SpliceAid2 [26], which enable accurate prediction and mapping of known splicing factor binding sites.

The following calculation parameters were chosen for SFmap:

Scoring function: COS(WR);
Medium stringency (Threshold [Significant] at p-value<0.005; [Suboptimal] at p-value<0.05);
Window size: 50.

## Supporting Information

Figure S1 **Minigene splicing assay of BRCA1 exon 9 and 10.** **A**. The pB1 wild type (WT) version of the minigene is shown. PCMV = promoter of the pCDNA3 vector. ATG = start codon. TAG = stop codon. +3C = insertion of cytosine as the third nucleotide in exon 8. pA = poly A signal. 1 = exon 1 of the alfa globin gene. BRCA1 exons from 8 to 12 are numbered. The black solid line represents introns. Dotted lines show alternative splicing of exon 9 and 10. **B**. Detection of BRCA1 splicing isoforms FL (inclusion of exon 9 and 10); D9 (skipping of exon 9); D10 (skipping of exon 10); D9,10 (skipping of exon 9 and 10).
(TIF)

Figure S2 **BRCA1 alignments between nine eutherian mammals.** The sequence of exon 11 critical region is in blue. Black characters represent the last 6 nucleotides of intron 10. The alternative donor site (5′ss) in exon 11 which gives rise to the Δ(11q) isoform is in white characters. Red are the nucleotide variations with respect to the human sequence. Ca. = Canis_familiaris; Eq. = Equus_caballus; Bo. = Bos_taurus; Ho. = Homo_sapiens; Pa. = Pan_troglodytes; Po. = Pongo_pygmaeus; Ma. = Macaca_mulatta; Mu. = Mus_musculus; Ra. = Rattus_norvegicus. Splicing regulatory proteins (predicted with SFmap) putatively binding to the human sequence are shown at the top of each sequence. The one corresponding to splicing regulatory motifs that are most conserved are highlighted. The list of putative sequences for splicing regulatory proteins and relative scores as reported in SFmap are listed.
(DOC)

Figure S3 Splicing factors predicted to bind. The sequence of deletion 2 and deletion 3 region are shown. Splicing factors predicted (by SFmap and/or SpliceAid) to bind these regions are listed.
(XLS)

Table S1 Information about all synonymous variants tested. The table includes the amino acid number, the nucleotide change, the dbSNP rs number, genomic location in hg19 coordinates (Chr 17 position),.PMID (for existing PubMed records) and our interpretation of the effects caused by the variations on different splicing events tested (isoform regulation).
(XLS)
