# Supplementary figures and images for: Evolutionary Constraint Helps Unmask a Splicing Regulatory Region in BRCA1 Exon 11
Source: PLoS One. 2012 May 16;7(5):e37255. doi: 10.1371/journal.pone.0037255 (PMC3353946; doi:10.1371/journal.pone.0037255)

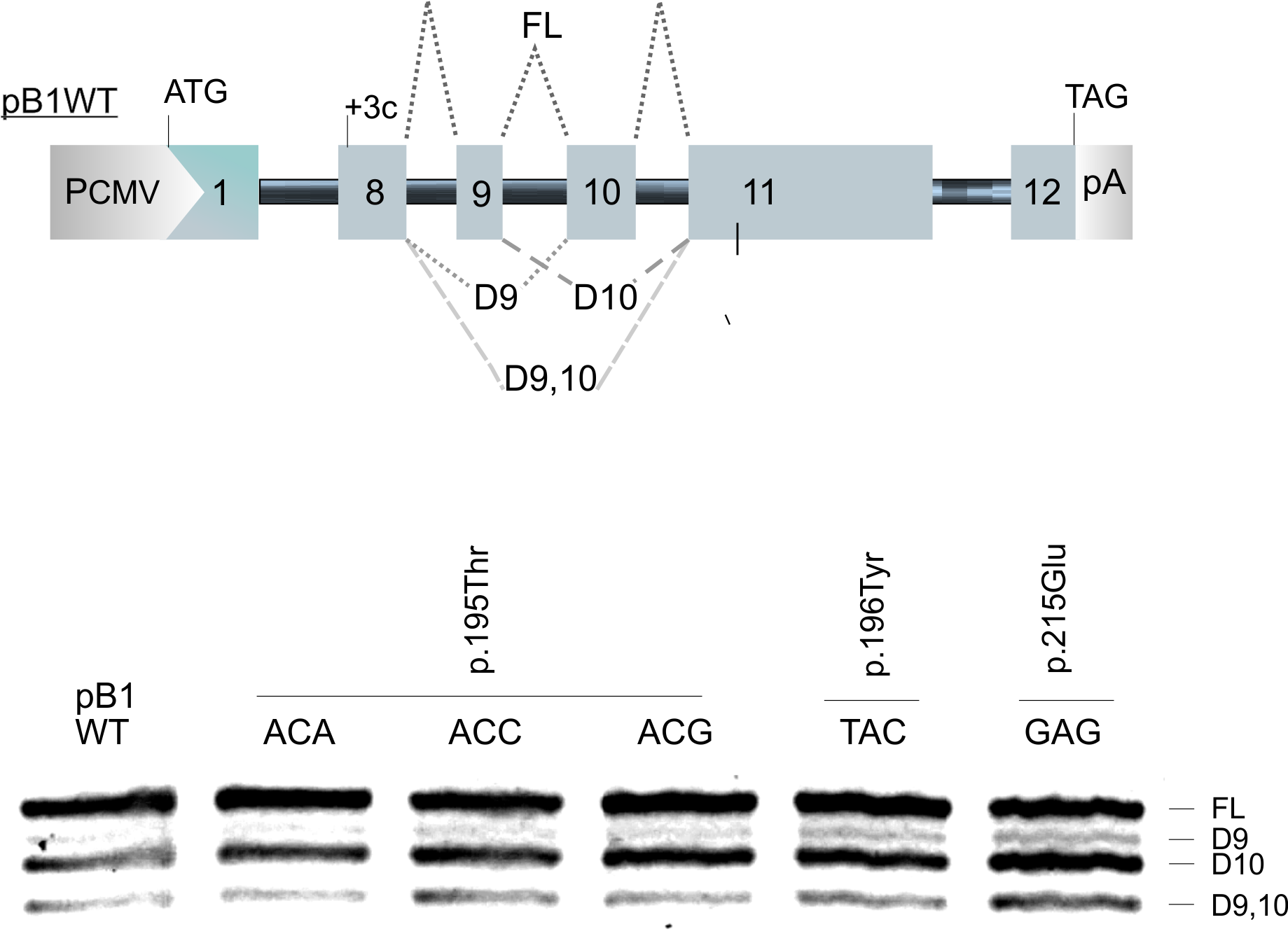

Supplement: Figure S1 — Minigene splicing assay of BRCA1 exon 9 and 10. A. The pB1 wild type (WT) version of the minigene is shown. PCMV = promoter of the pCDNA3 vector. ATG = start codon. TAG = stop codon. +3C = insertion of cytosine as the third nucleotide in exon 8. pA = poly A signal. 1 = exon 1 of the alfa globin gene. BRCA1 exons from 8 to 12 are numbered. The black solid line represents introns. Dotted lines show alternative splicing of exon 9 and 10. B. Detection of BRCA1 splicing isoforms FL (inclusion of exon 9 and 10); D9 (skipping of exon 9); D10 (skipping of exon 10); D9,10 (skipping of exon 9 and 10). (TIF) [file pone.0037255.s001.tif]
