# Supplementary material for: Evolutionary Constraint Helps Unmask a Splicing Regulatory Region in BRCA1 Exon 11
Source: PLoS One. 2012 May 16;7(5):e37255. doi: 10.1371/journal.pone.0037255 (PMC3353946; doi:10.1371/journal.pone.0037255)
Supplement: Figure S2 — BRCA1 alignments between nine eutherian mammals. The sequence of exon 11 critical region is in blue. Black characters represent the last 6 nucleotides of intron 10. The alternative donor site (5′ss) in exon 11 which gives rise to the Δ(11q) isoform is in white characters. Red are the nucleotide variations with respect to the human sequence. Ca. = Canis_familiaris; Eq. = Equus_caballus; Bo. = Bos_taurus; Ho. = Homo_sapiens; Pa. = Pan_troglodytes; Po. = Pongo_pygmaeus; Ma. = Macaca_mulatta; Mu. = Mus_musculus; Ra. = Rattus_norvegicus. Splicing regulatory proteins (predicted with SFmap) putatively binding to the human sequence are shown at the top of each sequence. The one corresponding to splicing regulatory motifs that are most conserved are highlighted. The list of putative sequences for splicing regulatory proteins and relative scores as reported in SFmap are listed. (DOC) [file pone.0037255.s002.doc]

FOX1 .

CUG-BP . CUG-BP CUG-BP YB1 .

MBNL . NOVA1

SC35 SRp20 . SC35 .

Ho. TTTCAGCTGCTTGTGAATTTTCTGAGACGGATGTAACAAATACTGAACATCATCAACCCAGTAATAATGATTTGAACACCACTGAGA

Ca. TTTCAGCTGCTTGTGAATTTTCTGGGGA---CATAACAAATATTGAACATCATCAATCCGGTAATAAAGATTTGACCACCACTGAGA

Eq. TTTCAGCTGCTTGTGAGTTTTCTGAGAAGGCCATAACAAATATTGAACATCATCAATCCAGTAATAAAGATTTGACCACCACTGAGA

Bo. TTCCAGCTGCTTGTGAGTTTTCTGAGAAGGACATAACAAATACTGAACATCATCAACTCAGTATTAAAGATCTGATCACCACCCAGA

Pa. TTTCAGCTGCTTGTGAATTTTCTGAGACGGATGTAACAAATACTGAACATCATCAACCCAGTAATAATGATTTGAACACCACTGAGA

Po. TTTCAGCTGCTTGTGAATTTTCTGAGACAGATGTAACAAATACTGAACATCATCAACCCAGTAATAACGATTTGAACACCACTGAGA

Ma. TTTCAGCTGCTTGTGAATTTTCTGAGAAGGATATAACAAATACTGAACATCATCAGGCCAGTAATAATGATTTGAACACCACTGAGA

Mu. TTTCAGCTGCTTGTGAGTTTTCTGAGGG---CATAAGAAACATTGAACATCATCAATGCAGTGA---TGATTTAAACCCTACTGAGA

Ra. TTTCAGCTGCTTGTGACTTTTCTGAGGG---CATAAGAAACATTGAACATCATCAATGCAGTGATAAAGATTTGAACCCCACTGAGA

FOX1 .

CUG-BP .

SRp55 .

Tra2b. Tra2b . FOX1 .SF2ASF. NOVA1

Ho. AGCGTGCAGCTGAGAGGCATCCAGAAAAGTATCAGGGTAGTTCTGTTTCAAACTTGCATGTGGAGCCATGTGGCACAAATACTCATG

Ca. AGCATGCAACTAAGAAGCATCCAGAAAAGTATCAGGGTATTTCTGTTTCAAACTTGCATGTGGAGCCATGTGGCACAAATACTCATG

Eq. AGCATGCAGCTGAGAAGCATCCAGAAAAGTATCAGGGTATTTCTGTTTCAAACTTGCATGTGGAGCCATGTGGCACAAATACTCATG

Bo. AGCATGCAACTGAGACGCATCCAGAAAAGTATCAGGGTATTTCTGTTTCAGACTTTCACGTGGAGCCATGTGGCACAGATACTCATG

Pa. AGCGTGCAACTGAGAGGCATCCAGAAAAGTATCAGGGTAGTTCTGTTTCAAACTTGCATGTGGAGCCATGTGGCACAAATACTCATG

Po. AGCGTGCAACTGAGAGGCATCCAGAAAAGTATCAGGGTAGTTCTGTTTCAAACTTGCATGTGGAGCCATGTGGCACAAATACTCATG

Ma. AACATGCAACTGAGAGGCATCCAGAAAAGTATCAGGGTAGTTCTGTTTCAAACTTGCATGTGGAGCCATGTGGCACAAATACTCATG

Mu. ATCATGCAACTGAAAGGCATCCAGAAAAATGTCAGAGTATTTCTATTTCAAATGTGTGTGTGGAGCCATGTGGCACAGATGCTCATG

Ra. ATCATGCAACTGAAAGGCATCCAGAAAAATGTCCGCGTATTTCTGTTGCAAACGTGCACGTGGAGCCGTGTGGCACAGATGCTCGTG

FOX1 .

NOVA1 Tra2b.

hnRNPAB hnRNPAB hnRNPA1 Tra2a .

SRp55. 9G8. SF2ASF. CUG-BP CUG-BP

Ho. CCAGCTCATTACAGCATGAGAACAGCAGTTTATTACTCACTAAAGACAGAATGAATGTAGAAAAGGCTGAATTCTGTAATA

Ca. CCAGCTCATTACAGCATGAGAACAGCAGTTTATTACTCACTAAACACAGAATGAATGTAGAAAAGGCTGAAATCTGTAATA

Eq. CCAGCTCATTGCAGCATGAGAACAGCAGTTTATTACTCACTAAAGACAGAATGAATGTAGAAAAGGCTGAATTCTGTAATA

Bo. CCAGCTCATTACAGCATGAGAACAGCAGTTTATTGCTCACTGAAAACAGACTGAATGTAGAAAAGGCTGAATTCTGTAATA

Pa. CCAGCTCATTACAGCATGAGAACAGCAGTTTATTACTCACTAAAGACAGAATGAATGTAGAAAAGGCTGAATTCTGTAATA

Po. CCAGCTCATTACAGCATGAGAACAGCAGTTTATTACTCACTAAAGACAGAATGAATGTAGAAAAGGCTGAATTCTGTAATA

Ma. CCAGCTCATTACAGCATGAGAACAG---TTTATTACTCACTAAAGACAGAATGAATGTAGAAAAGGCTGAATTCTGTAATA

Mu. CCAGCTCATTACAGCCTGAGACCAGCAGTTTATTGCTCATTGAAGACAGAATGAATGCAGAAAAGGCTGAATTCTGTAATA

Ra. CCAGCTCATTACAGCGTGGGACCCGCAGTTTATTGTTCACTGAGGACAGACTGGATGCAGAAAAGGCTGAATTCTGTGATA

Splicing Factor: SF2ASF, Motif: ugrwgvh, Cutoff: 0.6429

Sequence Position Genomic Coordinate K-mer Score

148 chr17:41246736 uggagcc 0.813

230 chr17:41246654 uguagaa 0.736

Splicing Factor: 9G8, Motif: acgagagay, Cutoff: 0.5000

Sequence Position Genomic Coordinate K-mer Score

213 chr17:41246671 acuaaagac 0.572

217 chr17:41246667 aagacagaa 0.585

Splicing Factor: SC35, Motif: gryymcyr, Cutoff: 0.6000

Sequence Position Genomic Coordinate K-mer Score

74 chr17:41246810 gaacacca 0.644

Splicing Factor: SC35, Motif: ugcygyy, Cutoff: 0.5857

Sequence Position Genomic Coordinate K-mer Score

5 chr17:41246879 agcugcu 0.741

Splicing Factor: Tra2alpha, Motif: gaagaggaag, Cutoff: 0.5000

Sequence Position Genomic Coordinate K-mer Score

234 chr17:41246650 gaaaaggcug 0.631

Splicing Factor: Tra2beta, Motif: gaagaa, Cutoff: 0.6500

Sequence Position Genomic Coordinate K-mer Score

231 chr17:41246653 guagaa 0.709

Splicing Factor: Tra2beta, Motif: aaguguu, Cutoff: 0.5000

Sequence Position Genomic Coordinate K-mer Score

87 chr17:41246797 aagcgug 0.500

114 chr17:41246770 aaguauc 0.557

Splicing Factor: SRp20, Motif: wcwwc, Cutoff: 0.7200

Sequence Position Genomic Coordinate K-mer Score

47 chr17:41246837 acauc 0.906

50 chr17:41246834 ucauc 0.917

53 chr17:41246831 ucaac 0.825

Splicing Factor: SRp55, Motif: yrcrkm, Cutoff: 0.7167

Sequence Position Genomic Coordinate K-mer Score

92 chr17:41246792 ugcagc 0.804

184 chr17:41246700 uacagc 0.750

Splicing Factor: hnRNPA1, Motif: uagaca, Cutoff: 0.5500

Sequence Position Genomic Coordinate K-mer Score

217 chr17:41246667 aagaca 0.667

Splicing Factor: hnRNPAB, Motif: auagca, Cutoff: 0.5500

Sequence Position Genomic Coordinate K-mer Score

185 chr17:41246699 acagca 0.656

196 chr17:41246688 acagca 0.664

Splicing Factor: MBNL, Motif: ygcuky, Cutoff: 0.6500

Sequence Position Genomic Coordinate K-mer Score

5 chr17:41246879 agcugc 0.802

8 chr17:41246876 ugcuug 0.812

Splicing Factor: NOVA1, Motif: ycay, Cutoff: 0.7500

Sequence Position Genomic Coordinate K-mer Score

50 chr17:41246834 ucau 0.839

153 chr17:41246731 ccau 0.932

170 chr17:41246714 ucau 0.953

180 chr17:41246704 ucau 0.932

Splicing Factor: CUG-BP, Motif: ugcug, Cutoff: 0.6000

Sequence Position Genomic Coordinate K-mer Score

5 chr17:41246879 agcug 0.771

8 chr17:41246876 ugcuu 0.771

20 chr17:41246864 uucug 0.766

41 chr17:41246843 uacug 0.671

92 chr17:41246792 ugcag 0.631

95 chr17:41246789 agcug 0.631

239 chr17:41246645 ggcug 0.618

246 chr17:41246638 uucug 0.725

Splicing Factor: YB1, Motif: caaccacaa, Cutoff: 0.5000

Sequence Position Genomic Coordinate K-mer Score

48 chr17:41246836 caucaucaa 0.542

Splicing Factor: FOX1, Motif: ugcaug, Cutoff: 0.4500

Sequence Position Genomic Coordinate K-mer Score

8 chr17:41246876 ugcuug 0.667

142 chr17:41246742 ugcaug 0.625

187 chr17:41246697 agcaug 0.583

226 chr17:41246658 ugaaug 0.627

Calculation parameters:

-----------------------------

Scoring function: COS(WR)

Significant p-value: 0.005

Suboptimal p-value: 0.05

Window size: 50
